# Planktonic ecological networks support quantification of changes in ecosystem health and functioning

**DOI:** 10.1038/s41598-023-43738-y

**Published:** 2023-10-04

**Authors:** Matteo Loschi, Domenico D’Alelio, Elisa Camatti, Fabrizio Bernardi Aubry, Alfred Beran, Simone Libralato

**Affiliations:** 1 Department of Life Sciences, University of Trieste, via Weiss 2, 34128 Trieste, Italy; 2 National Institute of Oceanography and Applied Geophysics – OGS, Trieste, Italy; 3 Department of Integrative Marine Ecology, Stazione Zoologica Anton Dohrn, Naples, Italy; 4 Institute of Marine Science (CNR ISMAR), National Research Council, Arsenale Tesa 104, Castello 2737/F, 30122 Venice, Italy

**Keywords:** Ecology, Ecological modelling, Ecological networks, Ecosystem ecology, Wetlands ecology

## Abstract

Plankton communities are the foundation of marine food webs and have a large effect on the dynamics of entire ecosystems. Changes in physicochemical factors strongly influence planktonic organisms and their turnover rates, making their communities useful for monitoring ecosystem health. We studied and compared the planktonic food webs of Palude della Rosa (Venice Lagoon, Italy) in 2005 and 2007. The food webs were developed using a novel approach based on the Monte Carlo random sampling of parameters within specific and realistic ranges to derive 1000 food webs for July of each year. The consumption flows involving Strombididae, *Evadne* spp. and *Podon* spp. were identified as the most important in splitting food webs of the July of the two years. Although functional nodes (FNs) differed both in presence and abundance in July of the two years, the whole system indicators showed very similar results. Sediment resuspension acted as a source of stress for the Venice Lagoon, being the most used resource by consumers while inhibiting primary producers by increasing water turbidity. Primary production in the water column was mainly generated by benthic FNs. Although the system was near an equilibrium point, it tended to increase its resilience at the expense of efficiency due to stress. This study highlights the role of plankton communities, which can serve to assess ecosystem health.

## Introduction

Plankton communities are the fundamental basis of marine food webs and drive the dynamics of entire ecosystems^1,2^. They are a complex group of organisms represented by different taxonomic categories, from Bacteria to fish larvae, that respond rapidly to both external influences and internal dynamics^3,4^. Environmental factors such as temperature, salinity, and pH affect taxa composition and productivity, and thus have strong impacts on plankton biodiversity^5^. In general, changes in physicochemical factors can strongly influence planktonic organisms and their turnover rates^6^, making their communities useful for monitoring ecosystem health^7–9^. For example, resulting changes in biomass can lead to changes in the trophic structure of the plankton community^10^. Overall, the plankton community is able to rapidly cope with new conditions^11,12^, in part due to a variety of processes and functions that can be performed by the community at the right time^13,14^. Some basal processes, such as mixotrophy, heterotrophy and detritivory, are more or less pronounced and may be expressed in response to changing environmental conditions, for example, to maintain system resilience^14^ and ecologically meaningful processes^13^.

Especially in transitional ecosystems such as a coastal lagoon, plankton populations are subject to fairly frequent and significant habitat disturbances, such as those caused by freshwater or seawater inputs^15,16^. In addition, the interplay of various forces, such as wave energy, fishing activities, atmospheric disturbances, and climate change, are among the most important factors influencing and determining the exchange of matter and energy between the system components, consequently affecting the presence of resident species or the exclusion and arrival of other species^17–19^.

Observing plankton communities can therefore inform on how and to what extent the aquatic ecosystem is able to cope with sources of variability, and it is crucial to understand what the key processes involved are^1,2,13^. In this context, ecological network models are useful tools since they provide estimates of flows that are difficult to disentangle and measure^14^. The analysis of plankton networks allows for a holistic understanding of changes in the functioning of the marine system, as they represent a wide range of different taxa involved in the basic processes of microbial loop and therefore link to fundamental trophic and ecological processes^13^. Applying such ecological network studies to coastal areas has a number of advantages, including the increased availability of knowledge and data^2^ and known high variability in responses of organisms in these systems due to multiple external pressures^20^.

The present study compares the trophic status of the Venice Lagoon in July 2005 and 2007, by developing plankton trophic networks. Using a novel approach based on iterative random samplings of parameters within specific realistic ranges, we reconstructed the planktonic food webs in July for three main reasons: (I) the unimodal annual peak of phytoplankton biomass occurs in this month^21^; (II) it is the period with the best biological data coverage from microzooplankton (size between 20 and 200 µm^22^) to mesozooplankton^23^ (size between 200 and 20,000 µm^22^); and (III) July has been historically characterised by some economically important ecological processes, such as recruitment of small pelagic fishes^24^. Therefore, our study aims to understand the planktonic community structure of the Venice Lagoon, a well-studied coastal system^25–27^, and to determine the role of specific functional groups within these communities, with the resulting potential implications at the food web level as an emerging effect of factors affecting the system at the two different times.

## Materials and methods

### Study area

The Venice Lagoon is one of the largest lagoons in the Mediterranean and is part of the Italian Long-Term Ecological Research Network (http://www.lteritalia.it/)^28^. In its history, this important transitional ecosystem has undergone relevant changes over time and has been extensively monitored over decades from different aspects due to its socio-ecological importance^29,30^. Plankton communities, especially phytoplankton and zooplankton, have also been monitored for many years, using different methods and approaches depending on different objectives or needs related to impact assessments^31–33^.

**Figure 1:**
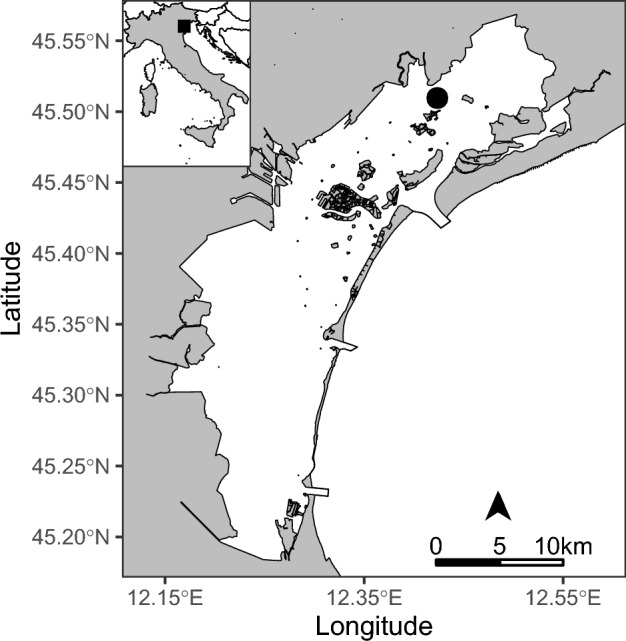
Location of Palude della Rosa (black circle) in the Lagoon of Venice (black square), Italy. This image was created using the sf^35^, ggplot2^36^, and ggsn^37^ packages (versions 1.0.9, 3.4.0, and 0.5.0 respectively) for R^38^.

The study focused on a limited shallow water area of the northern Venice Lagoon, i.e., the Palude della Rosa (Fig. 1), a typical lagoon area influenced by both freshwater inflow, from Canale Silone^34^, and saltwater input, due to incoming tides via Canale di Torcello^15^. Palude della Rosa covers an area of about 3.5 km^2^ with an average depth of about 0.5 m^34^ and is located in an intermediate position between the mainland coast and the sand barriers separating the Venice Lagoon from the Adriatic Sea. The plankton community at Palude della Rosa is therefore alternately influenced by river discharges and seawater intrusions^34^.

### Data

The available dataset covers July 2005 and 2007 and includes: taxonomic composition (where possible at species level or higher taxonomic levels), biomass (in mg C m^−3^) for organisms ranging in size from 1.150 to 28,000.000 µm. Plankton sampling was conducted during the neap tide to minimize variability associated with direct marine influence. Sampling and laboratory methods for biomasses of Bacteria, phytoplankton, mixoplankton, nanozooplankton, and microzooplankton are described in Pugnetti et al.^39^, while those of mesozooplankton, macrozooplankton, and non-living nodes in D’Alelio et al.^10^. Additional detailed description of methods used to collect plankton data, as well as ranges used for parameters and biomasses is reported in Supplementary Material.

### Structure of the plankton networks

The plankton was classified at the lowest possible taxonomic level. For a few organisms, species-level classification was possible, while for others only larger taxonomic groups were available. Data at the lowest taxonomic level were grouped into species with similar ecological functions, known interactions, and similar biological rates to simplify the model^40^, resulting in a set of ecologically meaningful functional nodes (FNs) (Table 1). Each FN is characterised by its size and trophic role. Although ecological preferences of plankton in the Venice Lagoon vary widely^41^, two macro-preferences (pelagic and benthic) were considered for all FNs. These categories were then used for the analyses.

**Table 1:** List of parameter values for simulations.

FN		Small description	Size (µm)	B (mg C m^−3^)		µ (d^−1^)		α (d^−1^)		ε		ph
				2005	2007	2005	2007	2005	2007	2005	2007	
1	Pico-phytoplankton	Mainly *Synechococcus* spp. (P)	1.150*	5.000	1.800	**0.912 (0.752; 1.045)**	**0.888 (0.752; 1.039)**	0.000	0.000	0.000	0.000	1.000
2	*Amphora exigua*	Pennate diatoms (B)	9.629*	–	0.481	–	**0.977 (0.819; 1.143)**	–	0.000	–	0.000	1.000
3	*Amphora* sp.	Pennate diatoms < 20 µm (B)	5.907*	–	1.424	–	**1.427 (1.173; 1.652)**	–	0.000	–	0.000	1.000
4	*Chaetoceros* spp.	Centric diatoms (P)	6.464*	–	0.583	–	**1.326 (1.103; 1.528)**	–	0.000	–	0.000	1.000
5	*Cocconeis scutellum*	Pennate diatoms (B)	10.904*	–	1.714	–	**0.940 (0.773; 1.075)**	–	0.000	–	0.000	1.000
6	*Cylindrotheca closterium*	Pennate diatoms (B)	6.701*	3.786	7.681	**0.967 (0.809; 1.145)**	**1.158 (0.959; 1.257)**	0.000	0.000	0.000	0.000	1.000
7	*Halamphora coffeaeformis*	Pennate diatoms (B)	12.291*	–	1.501	–	**0.889 (0.737; 1.006)**	–	0.000	–	0.000	1.000
8	*Navicula cryptocephala*	Pennate diatoms (B)	7.257*	0.534	0.243	**1.171 (0.984; 1.378)**	**1.198 (0.998; 1.391)**	0.000	0.000	0.000	0.000	1.000
9	*Navicula* spp.	Pennate diatoms > 20 µm (B)	8.094*	3.764	2.248	**1.108 (0.909; 1.289)**	**1.099 (0.905; 1.286)**	0.000	0.000	0.000	0.000	1.000
10	*Nitzschia frustulum*	Pennate diatoms (B)	4.925*	8.169	–	**1.599 (1.354; 1.896)**	–	0.000	–	0.000	–	1.000
11	*Nitzschia* sp.	Pennate diatoms < 20 µm (B)	3.856*	–	0.180	–	**1.933 (1.596; 2.257)**	–	0.000	–	0.000	1.000
12	*Psammodictyon panduriformis*	Pennate diatoms (P)	5.682*	–	0.290	–	**1.443 (1.194; 1.685)**	–	0.000	–	0.000	1.000
13	*Rhoicosphenia curvata*	Pennate diatoms (B)	7.376*	–	0.235	–	**1.205 (0.999; 1.397)**	–	0.000	–	0.000	1.000
14	*Thalassiosira* spp.	Centric diatoms (B)	9.087*	5.194	8.892	**1.036 (0.881; 1.202)**	**1.141 (0.943; 1.277)**	0.000	0.000	0.000	0.000	1.000
15	Pennate diatoms	Und. pennate diatoms > 10 µm (B)	6.765*	–	0.386	–	**1.259 (1.057; 1.489)**	–	0.000	–	0.000	1.000
16	Phyto-nanoflagellates	Und. Cryptophyceae (P)	4.579*	3.569	0.248	**1.107 (0.929; 1.305)**	**1.146 (0.952; 1.322)**	0.000	0.000	0.000	0.000	1.000
17	Phyto-nanoflagellates	Nanoflagellates (P)	3.000*	8.668	2.233	**1.418 (1.194; 1.650)**	**1.464 (1.201; 1.653)**	0.000	0.000	0.000	0.000	1.000
18	Mixo-dinoflagellates	Dinoflagellata < 20 µm (B)	9.968*	–	1.871	–	**0.846 (0.762; 0.903)**	–	**1.739 (1.539; 2.082)**	–	0.010	0.500
19	Hetero-dinoflagellates	Dinoflagellata (P)	31.309*	–	0.030	–	**0.629 (0.567; 0.665)**	–	**2.747 (2.515; 3.239)**	–	0.010	0.000
20	Hetero-ciliates	Ciliophora (Ciliata indet. < 20 µm) (B)	9.450*	–	0.003	–	**1.743 (1.461; 2.038)**	–	7.080	–	0.010	0.000
21	Hetero-ciliates	Ciliophora (Suctoria indet.) (B)	36.060*	–	0.007	–	0.735	–	3.525	–	0.010	0.000
22	*Mesodinium* cf. *rubrum*	95% auto ciliates (Ciliophora < 20 µm) (P)	9.450*	–	0.005	–	**0.760 (0.740; 0.796)**	–	**0.081 (0.077; 0.086)**	–	0.010	0.950
23	*Mesodinium* cf. *rubrum*	95% auto ciliates (Ciliophora) (P)	30.000*	0.064	–	**0.761 (0.742; 0.794)**	**–**	**0.082 (0.077; 0.086)**	–	0.010	–	0.950
24	Hetero-holotrich ciliates	Ciliophora (P)	30.000*	0.011	0.029	**0.778(0.675; 0.879)**	**0.785 (0.676; 0.892)**	**3.570 (2.898; 4.075)**	**2.697 (2.450; 3.261)**	0.010	0.010	0.000
25	Hetero-holotrich ciliates	Ciliophora (B)	70.000*	–	2.442	–	**0.774 (0.664; 0.868)**	–	**2.595 (2.446; 2.917)**	–	0.010	0.000
26	Hetero-hypotrich ciliates	Ciliophora (B)	55.560*	0.034	0.050	0.875	0.735	3.500	3.525	0.010	0.010	0.000
27	*Strombidinopsis* spp.	Hetero-choreotrich ciliates (Ciliophora) (P)	45.430*	–	2.071	–	**1.102 (0.988; 1.296)**	–	**2.474 (2.145; 2.925)**	–	0.010	0.000
28	Strombididae indet	30% mixo-oligotrich ciliates (Ciliophora) (P)	18.170*	0.118	0.003	**1.291 (1.159; 1.503)**	**1.269 (1.117; 1.503)**	**2.286 (1.902; 2.651)**	**2.163 (1.820; 2.567)**	0.010	0.010	0.300
29	Hetero-spirotrich ciliates	Ciliophora (P)	28.693*	1.360	1.133	**1.184 (1.078; 1.362)**	**1.199 (1.067; 1.404)**	**2.900 (2.457; 3.366)**	**2.633 (2.286; 3.076)**	0.010	0.010	0.000
30	Strombididae indet	30% mixo-oligotrich ciliates (Ciliophora) (P)	45.050*	–	0.213	–	**1.140 (0.979; 1.347)**	–	**1.936 (1.653; 2.235)**	–	0.010	0.300
31	Strobilididae indet	Hetero-choreotrich ciliates (Ciliophora) (P)	45.430*	–	0.150	–	**1.131 (0.980; 1.342)**	–	**2.553 (2.166; 2.978)**	–	0.010	0.000
32	*Tintinnopsis* spp.	Hetero-tintinnid ciliates (Ciliophora) (P)	19.700*	0.023	0.033	**1.294 (1.086; 1.488)**	**1.328 (1.130; 1.531)**	**5.564 (4.635; 6.406)**	**4.324 (3.890; 5.258)**	0.010	0.010	0.000
33	Hetero-tintinnid ciliates	Ciliophora (P)	49.610*	0.921	1.259	**1.298 (1.103; 1.462)**	**1.345 (1.142; 1.512)**	**5.470 (4.522; 6.327)**	**4.175 (3.791; 5.015)**	0.010	0.010	0.000
34	Hetero-nanoflagellates	(P)	3.000*	52.730	21.600	**0.883 (0.881; 0.887)**	**0.884 (0.881; 0.887)**	**1.779 (1.770; 1.789)**	**1.779 (1.769; 1.790)**	0.010	0.010	0.000
35	Meroplankton	Metazoa (micro-fraction) (P)	55.560*	0.034	0.700	**0.985 (0.632; 1.300)**	**1.027 (0.675; 1.328)**	**3.666 (2.065; 5.479)**	**2.821 (1.795; 3.825)**	0.200	0.200	0.000
36	Copepods juveniles	Metazoa (micro-fraction) (P)	65.870*	0.396	1.048	**1.118 (0.790; 1.371)**	**1.166 (0.802; 1.400)**	**4.449 (2.769; 6.273)**	**3.016 (2.014; 4.033)**	0.270	0.270	0.000
37	Copepods juveniles	Metazoa (meso-fraction) (P)	450.000	0.570	0.423	**0.290 (0.216; 0.353)**	**0.333 (0.271; 0.379)**	**1.187 (0.720; 1.587)**	**0.877 (0.683; 1.208)**	0.270	0.270	0.000
38	*Penilia avirostris*	Metazoa (Cladocera) (P)	800.000	0.025	**–**	**1.095 (0.782; 1.272)**	–	**4.929 (2.790; 6.954)**	–	**0.426 (0.367; 0.489)**	**–**	0.000
39	*Evadne* spp. & *Podon* spp.	Metazoa (Cladocera) (P)	900.000	0.003	0.012	**1.107 (0.801; 1.264)**	**1.154 (0.873; 1.303)**	**5.235 (2.924; 7.210)**	**2.900 (2.210; 4.332)**	**0.422 (0.361; 0.487)**	**0.415 (0.357; 0.478)**	0.000
40	*Paracalanus parvus complex*	Metazoa (Copepoda) (P)	850.000	0.045	0.059	**0.166 (0.107; 0.219)**	**0.119 (0.083; 0.149)**	**0.632 (0.378; 0.927)**	**0.311 (0.205; 0.424)**	**0.498 (0.472; 0.523)**	**0.493 (0.474; 0.518)**	0.000
41	*Acartia* spp.	Metazoa (Copepoda) (P)	891.000	2.195	0.801	**0.185 (0.133; 0.220)**	**0.135 (0.112; 0.150)**	**(0.744; 1.028)**	**0.355 (0.282; 0.493)**	**0.499 (0.476; 0.525)**	**0.490 (0.472; 0.516)**	0.000
42	*Centropages ponticus*	Metazoa (Copepoda) (P)	744.000	0.071	0.045	**0.174 (0.114; 0.219)**	**0.124 (0.085; 0.158)**	**0.663 (0.391; 0.928)**	**0.332 (0.222; 0.436)**	**0.501 (0.475; 0.526)**	**0.490 (0.471; 0.517)**	0.000
43	*Oithona* spp.	Metazoa (Cyclopoida) (P)	675.000	0.002	0.001	**0.074 (0.060; 0.085)**	**0.071 (0.059; 0.077)**	**0.351 (0.236; 0.472)**	**0.190 (0.155; 0.279)**	**0.293 (0.268; 0.322)**	**0.296 (0.269; 0.322)**	0.000
44	Carnivora	Metazoa (P)	28,000.000	0.704	0.666	0.007	0.007	**0.032 (0.023; 0.041)**	**0.023 (0.017; 0.034)**	**0.190 (0.184; 0.195)**	**0.190 (0.185; 0.195)**	0.000
45	Meroplankton	Larvae of Metazoa (Cirripedia, Gastropoda, Polychaeta, and Bivalvia) (P)	250.000	0.049	**–**	**0.363 (0.296; 0.399)**	–	**1.763 (1.199; 2.269)**	–	0.200	**–**	0.000
46	Meroplankton	Larvae of Metazoa (Cirripedia and Gastropoda) (P)	250.000	–	0.016	–	**0.378 (0.321; 0.406)**	–	**1.051 (0.831; 1.497)**	–	0.200	0.000
47	Decapods larvae	Metazoa (mainly Zoea) (P)	2044.000	0.169	**0.113 (0.066; 0.184)**	**0.353 (0.292; 0.395)**	**0.340 (0.277; 0.391)**	**1.657 (1.201; 2.161)**	**0.901 (0.770; 1.236)**	0.200	0.200	0.000
48	Harpacticoida	Metazoa (B)	728.000	0.054	0.025	**0.209 (0.147; 0.251)**	**0.178 (0.134; 0.206)**	**0.846 (0.516; 1.220)**	**0.743 (0.458; 1.036)**	**0.295 (0.268; 0.323)**	**0.293 (0.267; 0.322)**	0.000
49	Bacteria	Pico-hetero	1.150*	109.272	142.207	0.900	0.900	3.600	3.600	0.010	0.010	0.000
50	Small faecal pellets	Detritus	< 200.000	1.824	3.691	0.000	0.000	0.000	0.000	0.000	0.000	**–**
51	Large faecal pellets	Detritus	> 200.000	0.004	0.004	0.000	0.000	0.000	0.000	0.000	0.000	**–**
52	Particulate detritus	Detritus	–	316.534	330.632	0.000	0.000	0.000	0.000	0.000	0.000	**–**
53	Dissolved detritus	Detritus	–	5.026	4.163	0.000	0.000	0.000	0.000	0.000	0.000	**–**

Autotrophs, heterotrophs and mixotrophs have value of ph equal to 1, 0, and between 0 and 1, respectively. Median of parameters (in brackets the first and the third quartiles) estimated by the modelling approach are in bold, while the values that are not subject to change in regular font.

FN, functional node; *, equivalent sphere diameter (average); B, biomass; µ, rate of production per biomass unit; α, rate of consumption per biomass unit; ε, unassimilated fraction of  biomass consumed; (P),  pelagic; (B), benthic; ph, phototrophy proportion.

Four metabolic parameters were assigned to each FN: production rate per biomass unit (μ, as d^−1^), consumption rate per biomass unit (α, as d^−1^), unassimilated fraction of biomass consumed (ε, dimensionless), and the phototrophy proportion in individual metabolism (ph, dimensionless). The latter has a value of 0 for heterotrophs, 1 for autotrophs, and a value between 0 and 1 for mixotrophs. The metabolic parameters μ, α and ε have a range with a maximum and a minimum value as extreme values for each FN, depending on the specific metabolism of each FN, which in turn is influenced by water temperature^40^. The proportions of flows to non-living nodes (γ) describing the fate of faeces, mortalities, and excreta also have a range.

The ordinal qualitative trophic links between FNs are ranked with four different values: 0, 1, 2, 3, representing no interaction, weak interaction, moderate interaction, and strong interaction, respectively (Table 2). These values were determined based on expert knowledge of plankton trophic ecology.

**Table 2:** Ordinal qualitative interactions between functional nodes (FNs).

FNs	18	19	20	21	22	23	24	25	26	27	28	29	30	31	32	33	34	35	36	37	38	39	40	41	42	43	44	45	46	47	48	49
1	2	2	2	2			2		1		2	2	2	1	2	2	1	1	1		3	2										
2	2	2		2			2		2	2	2	2	2	2	2	2		2	2	2		2	3	2	1				2			
3	2	2		2			2		2	2	2	2	2	2	2	2		2	2	2		2	3	2	1				2			
4	2	2		2			2		2	2	2	2	2	2	2	2		2	2	2		2	3	2	1				2			
5	2	2		2			2	2	2	2	2	2	2	2	2	2		2	2	2		2	3	2	1	1			2			
6	2	2		2			2	2	2	2		2	2	2	2	2		2	2	2	3	2	2	2	1	1		2	2			
7	2	2		2			2	2	2	2		2	2	2	2	2		2	2	2		2	3	2	1	1			2			
8	2	2		2			2		2	2	2	2	2	2	2	2		2	2	2	3	2	2	2	1	1		2	2			
9	2	2		2			2		2	2	2	2	2	2	2	2		2	2	2	3	2	2	2	1	1		2	2			
10							2		2		2	2	2	2	2	2		2	2	2	3	2	2	2				2				
11	2	2	2	2			2		2		2	2	2	2	2	2		2	2	2		2	3	2					2			
12	2	2		2			2		2	2	2	2	2	2	2	2		2	2	2		2	3	2	1				2			
13	2	2		2			2		2	2	2	2	2	2	2	2		2	2	2		2	3	2	1				2			
14	2	2		2			2		2	2	2	2	2	2	2	2		2	2	2	3	2	2	2	1	1		2	2			
15	2	2		2			2		2	2	2	2	2	2	2	2		2	2	2		2	3	2	1				2			
16	2	2	2	2	3	3	2		2		2	2	2	2	2	2		2	2	2	3	2	2					2	2			
17	2	2	2	2			2		2		2	2	2	2	2	2		2	2	2	3	2	2					2	2			
18		2		2			2	2	2	2	2	2	2	2	2	2		2	2	2		2	3	2	3	2			2			
19		2		2			2	2	2	2				2		2			2	2		2	3	3	3	3			2			
20		2		2			2		2	2	2	2	2	2	2	2		2	2	2		2	3	2	3	2			2			
21		2		2			2	2		2				2		2			2			2	2	3	3	3			2			
22		2		2			2		2	2	2	2	2	2	2	2		2	2			2	3	2	3	2			2			
23							2		2							2			2		2	2	2	3	3	3		2				
24		2		2			1	2	2							2			2	2	2	2	2	3	3	3		2	2			
25																						3	2	3	3	3			2			
26																			1		1	3	1	3	3	3		1	2			
27								2											1			3	2	3	3	3			2			
28		2		2			2	2	2	2		1	2	2		2		2	2	2	2	2	2	2	3	3		2	2			
29		2		2			2	2	2	2			2	2		2			2	2	1	2	2	3	3	3		2	2			
30								2											1			3	2	3	3	3			2			
31								2											1			3	2	3	3	3			2			
32		2		2			2	2	2	2		1	2	2		1		2	2	2	2	2	2	2	3	3		2	2			
33								2											1		1	3	1	3	3	3		1	2			
34	1	1	1	1			2		2		2	2	2	2	2	2		2	2	2	3	2	2					2	2			
35																									3	2			2			
36																									3	2			2			
37																									1		3			2		
38																											3			2		
39																											3			2		
40																											3			2		
41																											3			2		
42																											3			2		
43																											3			2		
45																									2		3			2		
46																											3			2		
47																														2		
48																											3			2		
49	1	1	1	1	1	1	2		1		2	2	1	1	2	2	3	1	1													
50																										2					3	3
51																															3	3
52																								1							3	3
53																																3

Prey are in the rows and predators are in the columns. 1 = weak interaction, 2 = moderate interaction, 3 = strong interaction. Empty cells represent no interaction.

### Modelling approach

Plankton food webs were based on biomasses (B, as mg C m^−3^) of plankton FNs and flows between them as daily flows (mg C m^−3^ d^−1^).

Weighted plankton food webs have been developed that assume a balance between production, natural mortality, and consumption by predators for each living node k:

1 $${\upmu }_{\mathrm{k}}\cdot {\mathrm{B}}_{\mathrm{k}}-{\sum }_{\mathrm{j}=1}^{\mathrm{n}}\left({\mathrm{\alpha }}_{\mathrm{j}}\cdot {\mathrm{B}}_{\mathrm{j}}\cdot {\mathrm{DC}}_{\mathrm{k},\mathrm{j}}\right)-{\mathrm{m}}_{\mathrm{k}}=0$$

where μ_k_ is the production rate per biomass unit of FN k and B_k_ is its biomass. The first negative term is the sum of the consumptions of predator j as the product of the predator’s biomass B_j_, its consumption rate per unit of biomass α_j_, and the proportion of living prey in the predator’s diet (DC_k,j_). The total number of FNs in the network is n and m_k_ is the natural mortality of node k.

And for each non-living node d, a balance is established between flows to non-living nodes, consumption by detritivores, exports and imports:

2 $${\sum }_{\mathrm{i}=1}^{\mathrm{n}}\left[{\upgamma }_{\mathrm{i},\mathrm{d}}\cdot \left({\upvarepsilon }_{\mathrm{i}}\cdot {\mathrm{\alpha }}_{\mathrm{i}}\cdot {\mathrm{B}}_{\mathrm{i}}+{\mathrm{m}}_{\mathrm{i}}\right)\right]-{\sum }_{\mathrm{j}=1}^{\mathrm{n}}\left({\mathrm{\alpha }}_{\mathrm{j}}\cdot {\mathrm{B}}_{\mathrm{j}}\cdot {\mathrm{DC}}_{\mathrm{d},\mathrm{j}}\right)-{\mathrm{ex}}_{\mathrm{d}}+{\mathrm{im}}_{\mathrm{d}}=0$$

where γ_i,d_ is the proportion of flows from any node i to the non-living node d and ε_i_ is its unassimilated fraction of biomass consumed. The first negative term is the sum of the consumptions of detritivores j as the product of the predator’s biomass B_j_, their consumption rate per biomass unit α_j_, and the proportion of non-living nodes in the diet of the predator (DC_d,j_). The amount of export and import of node d are ex_d_ and im_d_, respectively.

For each FN (i), production (P = μ ∙ B), consumption (Q = α ∙ B), and unassimilated (UN = ε ∙ α ∙ B) were related to estimate respiration (R):

3 $${\mathrm{R}}_{\mathrm{i}}={\mathrm{Q}}_{\mathrm{i}}-{\mathrm{P}}_{\mathrm{i}}\cdot (1-{\mathrm{ph}}_{\mathrm{i}})-{\mathrm{UN}}_{\mathrm{i}}$$

where ph_i_ is the phototrophy proportion of node i. Each time a trophic network met all conditions and constraints, it was accepted and the process began again until the ensemble of 1000 networks was reached. This procedure was applied to both 2005 and 2007.

### Randomly generated networks with a-posteriori validity check

The system of equations was applied using an iterative approach in which μ, α, ε, and γ of each FN were randomly sampled from their range. The values obtained for the proportions of flows to non-living nodes were transformed so that the sum of proportions for each FN was equal to 1. Trophic links were transformed from ordinal qualitative values to quantitative values by randomly drawing two boundaries between 0 and 1 for each consumer. We constructed the matrix of the proportion of the diet (DC_ij_, with flows from prey i to predator j) as follows: ordinal qualitative values equal to 1 were replaced by random values sampled from 0 and the first boundary; ordinal qualitative values equal to 2 were replaced by random values sampled from the first and second boundaries; and ordinal qualitative values equal to 3 were replaced by random values sampled from the second boundary and 1. Finally, the values obtained were transformed so that the sum of proportions of trophic links for each consumer was equal to 1. All samplings to determine the proportions of flows to non-living nodes, metabolic parameters, and links were performed using a uniform distribution.

Equations (1) and (2) were used for all FNs of the food web to establish a system of algebraic linear equations in which several parameters had a range (μ, α, ε, and γ) or were defined only in ordinal qualitative terms (DC_ij_). Other parameters such as natural mortality, exports, imports, respirations, and gross food conversion efficiency were estimated using the previous parameters. Considering the range of parameters, the system of equations does not have a unique solution. To examine all potential parameter combinations when no relevant information about parameter distributions is available, we randomly sampled them from a uniform distribution over the specified ranges and analysed a posteriori the distribution of parameters for valid networks. The resulting networks were tested for ecological and thermodynamic realism based on a set of simple constraints to limit respiration and gross food conversion efficiency.

Thus for each FN (i):

4 $${\mathrm{R}}_{\mathrm{i}}\ge 0$$

and for each consumer (j):

5 $$0.15\le {\mathrm{GE}}_{\mathrm{j}}\le 0.5$$

where R_i_ and GE_j_ are respectively the respiration flow and gross food conversion efficiency^42–44^, the ratio of heterotrophic production to consumption ${\mathrm{P}}_{\mathrm{j}}\cdot (1-{\mathrm{ph}}_{\mathrm{j}})/{\mathrm{Q}}_{\mathrm{j}}$, of nodes i and j, respectively.

### Indicators

A set of whole system indicators and the omnivory index, which provide information on the ecological characteristics of food webs, were calculated (Table 3). Each indicator was calculated for the two studied plankton networks to obtain a distribution of values with which statistical tests were performed for comparison.

**Table 3:** Indicators for the study of the networks of the Venice Lagoon.

Indicators	Abbreviation	Formula	Brief description	References
Relative ascendency	A/C	$\frac{{\sum }_{\mathrm{i}=0}^{\mathrm{n}}{\sum }_{\mathrm{j}=1}^{\mathrm{n}+2}\left({\mathrm{T}}_{\mathrm{ij}}{\cdot \mathrm{log}}_{2}\frac{{\mathrm{T}}_{\mathrm{ij}}\mathrm{T}..}{{\mathrm{T}}_{\mathrm{i}.}{\mathrm{T}}_{.\mathrm{j}}}\right)}{- {\sum }_{\mathrm{i}=0}^{\mathrm{n}}{\sum }_{\mathrm{j}=1}^{\mathrm{n}+2}\left({\mathrm{T}}_{\mathrm{ij}}\cdot {\mathrm{log}}_{2}\frac{{\mathrm{T}}_{\mathrm{ij}}}{\mathrm{T}..}\right)}$	The proportion of the possible organisation that is actually realised in a system. It can take values between 0 (no efficiency and maximum resilience) and 1 (maximum efficiency and no resilience)	^45^
Relative internal ascendency	A_i_/C_i_	$\frac{{\sum }_{\mathrm{i}=1}^{\mathrm{n}}{\sum }_{\mathrm{j}=1}^{\mathrm{n}}\left({\mathrm{T}}_{\mathrm{ij}}{\cdot \mathrm{log}}_{2}\frac{{\mathrm{T}}_{\mathrm{ij}}\mathrm{T}..}{{\mathrm{T}}_{\mathrm{i}.}{\mathrm{T}}_{.\mathrm{j}}}\right)}{- {\sum }_{\mathrm{i}=1}^{\mathrm{n}}{\sum }_{\mathrm{j}=1}^{\mathrm{n}}\left({\mathrm{T}}_{\mathrm{ij}}\cdot {\mathrm{log}}_{2}\frac{{\mathrm{T}}_{\mathrm{ij}}}{\mathrm{T}..}\right)}$	The proportion of the possible organisation that is actually realised, calculated on the basis of the system's internal exchanges. It can take values between 0 (no efficiency and maximum internal resilience) and 1 (maximum internal efficiency and no resilience)	^46^
Finn’s cycling index	FCI	$\frac{{\sum }_{\mathrm{i}=1}^{\mathrm{n}}\left[\left(1-\frac{1}{{\mathrm{q}}_{\mathrm{ii}}}\right)\cdot {\mathrm{T}}_{\mathrm{i}}\right]}{{\sum }_{\mathrm{i}=0}^{\mathrm{n}}{\sum }_{\mathrm{j}=1}^{\mathrm{n}+2}{\mathrm{T}}_{\mathrm{ij}}}$	The fraction of the sum of flows that can be attributed to cycling. It can range from 0 (no recycling) to 1 (all matter/energy is recycled)	^47^
Ratio of detritivory to herbivory	D/H	$\frac{{\sum }_{\mathrm{j}=1}^{\mathrm{k}}{\sum }_{\mathrm{i}=\mathrm{k}+1}^{\mathrm{n}}{\mathrm{T}}_{\mathrm{ij}}}{{\sum }_{\mathrm{j}=1}^{\mathrm{k}}{\sum }_{\mathrm{i}=1}^{\mathrm{n}}\left({\mathrm{T}}_{\mathrm{ij}}\cdot {\mathrm{ph}}_{\mathrm{i}}\right)}$	The resource of the lowest trophic level is most used by the consumers of the system: if the quotient is greater than 1, it means that the non-living nodes are consumed more than the primary producers; if it is less than 1, the primary producers are consumed more; while if the quotient is about 1, the non-living nodes and the primary producers are consumed to a similar extent	^48^
Ratio of primary production to community respiration	PP/R	$\frac{{\sum }_{\mathrm{i}=1}^{\mathrm{n}}\left({\mathrm{P}}_{\mathrm{i}}\cdot {\mathrm{ph}}_{\mathrm{i}}\right)}{{\sum }_{\mathrm{i}=1}^{\mathrm{n}}{\mathrm{R}}_{\mathrm{i}}}$	In the early stages of an ecological succession it is higher or lower than 1, while in the mature stages it is about 1	^49^
Omnivory index	OI	$\sum_{\mathrm{i}=1}^{\mathrm{n}}\left\{{\left[{\mathrm{TL}}_{\mathrm{i}}-\left({\mathrm{TL}}_{\mathrm{j}}-1\right)\right]}^{2}\cdot {\mathrm{DC}}_{\mathrm{ij}}\right\}$	The variance of trophic levels of a consumer's prey. If the consumer feeds on many trophic levels, it takes on a large value, whereas if the consumer is specialized, it takes on 0	^50^

For each of these indicators, the abbreviation, the formula and a brief description of its meaning are given.

### Statistical analysis

Principal component analysis (PCA) was carried out on the consumption flows. Only the non-constant flows shared by the food webs of the July of two years were selected, since they had a different number of nodes. Because the value ranges of the variables were very different, these were shifted to be zero-centered and scaled to have unit variance before analysis to avoid larger value variables dominating the PCA results. In analysing the PCA results, the first 15 loadings, ranked by their relative importance to the first two principal components, were considered to determine the FNs with the greatest contribution to the split of the simulations of the July of the two years tested in terms of consumption flows.

Comparisons between 2005 and 2007 food webs and between pelagic and benthic primary production within July of each year were made using Mood's median test. The one-sample sign test was used to verify if the values of relative ascendency were statistically different from a reference value of 0.4596^51^. Non-parametric tests were used because the assumptions for parametric ones were not met.

The entire modelling approach, including the calculation of indicators and statistical analyses, was developed in R version 4.2.2^38^ with RStudio version 2022.07.2 + 576^52^, using EnvStats^53^, DescTools^54^, and NetIndices^55^ packages versions 2.7.0, 0.99.47, and 1.4.4.1, respectively.

## Results

### Principal component analysis

**Figure 2:**
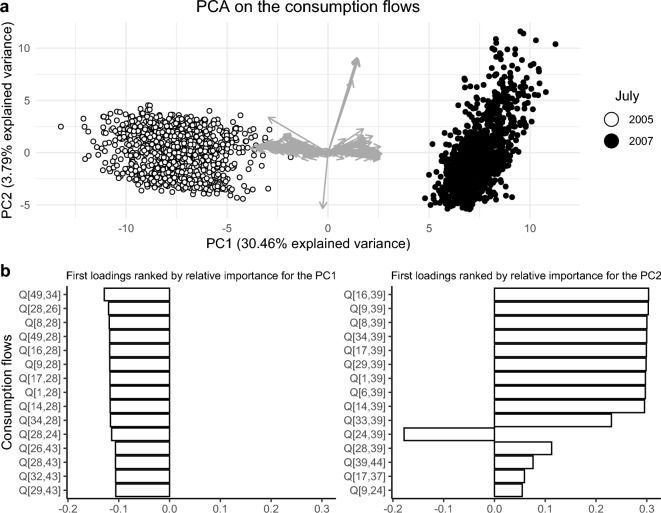
Principal component analysis. The plot was created using the ggplot2^36^ and ggpubr^56^ packages, versions 3.4.0 and 0.6.0, respectively, for R^38^. (**a**) The first two principal components are given and together account for 34.251% of the total variance. (**b**) The first 15 loadings ranked by their relative importance to the first two principal components, are given. The consumption flows involving FN 28 (Strombididae) contribute most to the first principal component, while those involving FN 39 (*Evadne* spp. and *Podon* spp.) contribute most to the second principal component. "Q" indicates the consumption flow moving from the prey (the first number in brackets) to the predator (the second number).

PCA on the consumption flows shows that the first two principal components together account for 34.251% of the total variance (Fig. 2a). The first 15 loadings, ranked by their relative importance for the first two principal components, are shown in Fig. 2b. The consumption flows involving FN 28 (Strombididae) contribute most to the first principal component, while the consumption flows involving FN 39 (*Evadne* spp. and *Podon* spp.) contribute most to the second principal component.

### Shifting topological roles in key functional nodes

**Figure 3:**
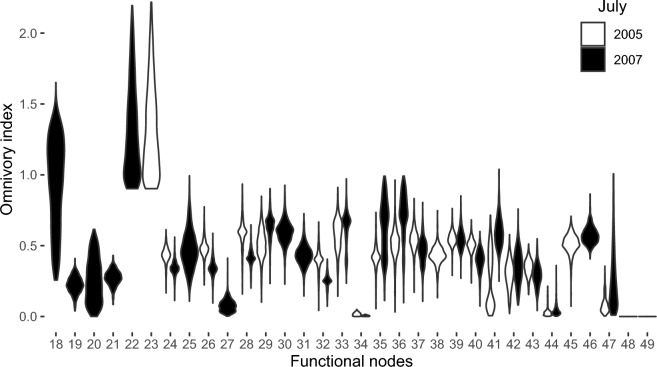
Violin plot of the omnivory index (OI) for each consumer in July in each year (2005 and 2007). Each violin was plotted to have the same maximum width, but if there is only one violin in a year, it has twice the maximum width. The plot was created using the ggplot2 package^36^ version 3.4.0 for R^38^.

The results show that consumers with the phototrophy proportion greater than or equal to 0.5 have higher OI values, namely mixo-dinoflagellates and *Mesodinium* cf. *rubrum* (FNs 18, 22, and 23). On the other hand, exclusive detritivores, namely Harpacticoida and Bacteria (FNs 48 and 49, respectively), have the lowest value, i.e., zero (Fig. 3).

### Output structure of the models

The iterative process to develop ecological networks using the input data sets (and ranges) on plankton communities in the Venice Lagoon for the July of 2005 and 2007 resulted in 1000 meaningful networks for each year. These valid networks result from a random selection of parameters within the ranges and do not lead to unrealistic ecological processes (such as unrealistic respiration, mortality, etc.). The first, second, and third quartiles, listed in Table 1, are used to describe the results of the parameters since they do not have a normal distribution.

The median primary production of pelagic and benthic nodes was respectively 20.890 and 27.144 mg C m^−3^ d^−1^ for 2005, and 6.373 and 28.347 mg C m^−3^ d^−1^ for 2007. Mood's median test between pelagic and benthic primary production was highly significant in July of each year (the p-value was 0 in July of both years and the value of the z-statistic was − 31.029 for 2005 and − 44.710 for 2007). The results of Mood's median test for total imports to undissolved detritus and for parameters related to FNs 28 (Strombididae) and 39 (*Evadne* spp. and *Podon* spp.) between 2005 and 2007 are shown in Table 4. Compared to 2005, total imports to undissolved detritus (FNs 50, 51, 52) increased in 2007, OI and percent consumption of FN 28 prey that can provide "domesticable" plastids, i.e., phyto-nanoflagellates, mixo-dinoflagellates, and *Mesodinium* cf. *rubrum* smaller than 20 µm^57^, which are FN 16, 17, 18, and 22, decreased in the diet of FN 28, while the production rate per biomass unit and OI of FN 39 remained the same.

**Table 4:** The results of all comparisons between 2005 and 2007 are given.

Variable	z	*p* value	2005 Median	2007 Median
Import to undissolved detritus (mg C m^−3^ d^−1^)	− 40.776	0	205.379	258.444
μ of FN 28 (d^−1^)	0.894	0.371	1.291	1.269
Q of FN 28 prey that can provide “domesticable” plastids (%)	37.825	0	22.184	17.474
OI of FN 28	34.429	0	0.597	0.412
OI of FN 39	1.341	0.180	0.547	0.541
A/C	− 5.097	3.452⋅10^–7^	0.435	0.437
A_i_/C_i_	44.442	0	0.419	0.384
FCI	− 39.971	0	0.061	0.088
D/H	34.606	0	34.778	25.564
PP/R	44.710	0	0.139	0.083

“z” is the value of Mood’s median test.

μ, production rate per biomass unit; FN, functional node; Q, consumption; OI, omnivory index; A/C, relative ascendency; A_i_/C_i_, relative internal ascendency; FCI, Finn’s cycling index; D/H, ratio of detritivory to herbivory; PPR, ratio of primary production to community respiration.

### Whole system indicators

**Figure 4:**
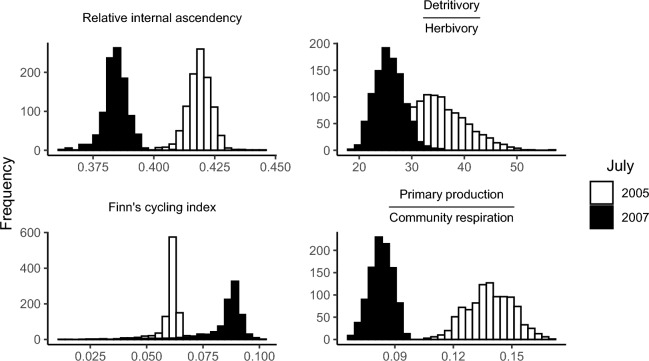
Histograms of some whole system indicators for July of the two years (2005 and 2007): relative internal ascendency (A_i_/C_i_), ratio of detritivory to herbivory (D/H), Finn's cycling index (FCI), and ratio of primary production to community respiration (PP/R). Graphs were generated using the ggplot2 package^36^ version 3.4.0 for R^38^.

The results of the comparison of each whole system indicator between the July of 2005 and 2007, calculated using Mood's median test, are shown in Table 4. For each whole system indicator, the test revealed significant statistical differences between July of each year. Median values of Finn's cycling index and relative ascendency increased over time, while median values of relative internal ascendency, detritivory to herbivory ratio, and primary production to community respiration ratio decreased. The distributions of some whole system indicators are shown in Fig. 4.

## Discussion

This study focused on modelling summer plankton food webs in one of the most important transitional ecosystems of the Mediterranean, the Venice Lagoon. The structure of the plankton food web was numerically derived in July 2005 and 2007 based on experimental data. In this ecosystem, the plankton community is highly influenced by a mixed control mechanism that depends mainly on tidal conditions affecting salinity and nutrient gradients, but also on anthropogenic influences^29,30^, that may limit the approach used. Nonetheless, the choice of the same month and the sampling carried out during the neap tide seem to have minimized the environmental variability, which is supported by the fact that the whole system indicators associated with the networks of July of the two years largely converge despite the differences in the composition and abundance of the FNs.

The results show that the consumption flows involving FNs 28 and 39 were the most important in differentiating the food webs of the two years. These FNs are mixotrophic ciliates (Strombididae, unicellular facultative mixotrophs) and Cladocera (i.e., *Evadne* spp. and *Podon* spp., fast-growing metazoans), respectively. Strombididae feed on small unicellular organisms belonging to the pico- and nanoplankton, of which some photosynthetic ones are retained as plastids for photosynthesis, while the heterotrophic ones are digested for energy production^57^. Hence, Strombididae are called generalist non-constitutive mixotrophs^58^. In 2007, there was a greater diversity of autotrophic prey of FN 28 (Strombididae), resulting in a decrease in OI compared to 2005. However, the percent consumption of its prey that provide "domesticable" plastids^57^, i.e., phyto-nanoflagellates (FNs 16 and 17), mixo-dinoflagellates (FN 18), and *Mesodinium* cf. *rubrum* smaller than 20 µm (FN 22), decreased and its production rate per biomass unit did not change in July of the two years (Table 4). The combination of these factors may indicate a more heterotrophic behaviour of Strombididae in 2007 by increasing predation on other, more abundant, unicellular prey such as Bacteria (FN 49) or hetero-nanoflagellates (FN 34) (Table 1).

In 2007, even hetero-nanoflagellates were an important food for some metazoans (Table 2), such as *Evadne* spp. and *Podon* spp. (FN 39), whose biomass increased by an order of magnitude in 2007 (Table 1). Cladocerans are organisms with an affinity for marine and coastal waters and hardly reside in the interior of the Venice Lagoon^23,59^, but under certain tidal conditions, incoming marine waters cause them to extend into more interior areas of the lagoon^59^, such as Palude della Rosa. Cladocerans are strongly influenced by the seasonality and spatial variability of environmental conditions^60^, thus parthenogenesis allows them to respond very quickly to environmental changes^61^. Their higher growth rates, compared to other planktonic crustaceans, must be sustained by higher consumption rates of prey whose abundance is more stable over time^61^, such as picoplankton^61,62^ (size between 0.2 and 2 µm^22^). This ability to respond rapidly to environmental variability was also evident in our study. In fact, a greater number of FNs, with a lower abundance (Table 1), fulfilled the function of primary producers in 2007. Thus *Evadne* spp. and *Podon* spp. (FN 39) doubled the number of prey, from 16 in 2005 to 32 in 2007, to maintain their high growth rate, while their OI remained constant (Table 4). Although the abundance of these crustaceans was very low (Table 1), their presence under certain conditions may reveal structural and functional changes already observed in other coastal planktonic food webs^10^.

Despite differences in community composition, due to interannual population fluctuations typical of transitional environments such as coastal lagoons^63,64^, whole system indicators show very similar results for the planktonic food web for July of both years. In our models, an inverse trend is observed between FCI and A_i_/C_i_, with median FCI higher and median A_i_/C_i_ lower in 2007 than in 2005 (Table 4). Although FCI is considered an indicator of system maturity^49^, it can be used in conjunction with other whole system indicators, such as A_i_/C_i_, to assess whether or not the system is stressed^46^. Although FCI is a partial measure of recycling in networks^65^, it is used here to compare results with other studies. However, the use of a whole system indicator that provides more accurate information about recycling within the system, such as the comprehensive cycling index^65^, is strongly recommended for future studies.

Our results indicate a significant increase in total import to the undissolved detritus (FNs 50, 51, and 52) from 2005 to 2007 (Table 4), which is confirmed by sampling of their total biomass, which increased from 318.362 in 2005 to 334.327 mg C m^−3^ in 2007 (Table 1). This led to a progressive increase of stress in the system, which increased matter/energy recycling and, consequently, FCI^66^. At the same time, A_i_/C_i_ decreased so that resilience increased and the system could cope with the perturbation^66^. The efficiency associated with internal flows decreased so that the system became more dependent on external flows^46^. Systems with lower A_i_/C_i_ ratios are more resilient because they have higher redundancy in trophic pathways, which allows them to recover disrupted ones^46^. However, systems with lower A_i_/C_i_ ratios are not resistant because they have low internal stability, which makes them more susceptible to external influences that can alter their configuration^46^.

In our models, A/C increased even if A_i_/C_i_ decreased over time, so overall efficiency increased even if internal efficiency decreased. The A/C median values below 0.5 in July of both years indicate that the overall resilience of the system under study was greater than its overall efficiency^45^. In particular, for ecological networks, an A/C value of 0.4596 has been suggested as optimal^51^ to represent two opposing trends in a system development, efficiency and resilience^45^. The A/C medians are statistically lower than the optimum, suggesting that the system likely increased its resilience at the expense of its efficiency to cope with the source of stress. This tendency of A/C is similar to eutrophication^67^ but the enrichment comes from organic matter rather than nutrients. The system should have increased its efficiency to improve its sustainability (robustness) in the long term^51^.

The Venice Lagoon is an ecosystem exposed to various natural and anthropogenic influences^68^. In particular, during the first decade of the 2000s, several factors led to the resuspension of sediments: dredging of new large channels, increasing number and speed of boats^69^, lower stabilisation of sediments due to the decline of seagrass^70,71^, and widespread use of mechanical fishing gears for harvesting Manila clams (*Ruditapes philippinarum*)^71,72^.

Sediment resuspension led to an increase in water turbidity and consequently to a decrease in phytoplankton^31^. In this regard, chlorophyll *a*, which is a proxy for phytoplankton biomass, showed a decreasing trend from 2001 to 2007^21^. Our work also showed that the PP/R values were well below 1 in July of both years, suggesting that sediment resuspension simultaneously inhibited primary producers due to turbidity and favoured detritivores due to the increase in organic carbon content in the water column^72^. As a result, community respiration increased (respiration of Bacteria, FN 49, has medians of 84.187 and 90.905% of community respiration in 2005 and 2007, respectively). Similarly, hetero-nanoflagellates (FN 34), which fed primarily on Bacteria (Table 2), seemed to benefit from this condition, contributing to a significant increase in community respiration (median values of 13.367 and 4.537% of community respiration in 2005 and 2007, respectively). Among the mesozooplanktonic FNs, which are between 200 and 20,000 µm in size^22^, a similar reasoning could be applied to *Acartia* spp. (FN 41), as in both years their biomass alone was greater than the median of the sum of the other mesozooplanktonic FNs (Table 1).

In the Venice Lagoon, intense sediment resuspension from Manila clam harvesting was a source of stress^73^ that acted like an external press perturbation^74^ on the system. In particular, our results confirm that detrital resuspension was a necessary component for the Venice Lagoon ecosystem^75,76^. In fact, our models estimate a mandatory import to non-living nodes to maintain detritivore consumption. The very high values of D/H make it clear that non-living nodes were the most utilised resource at the lowest trophic level. Nevertheless, only 4 FNs performed the detritivore function (Table 2): *Acartia* spp. (FN 41), *Oithona* spp. (FN 43), Harpacticoida (FN 48), and Bacteria (FN 49). Of these, Bacteria benefited the most from this condition^17^, because in both years their biomass alone was higher than the sum of the other FNs, making the decomposition processes significant^29^. Thanks to sediment resuspension, heterotrophic Bacteria were able to maintain high densities even when the carbon source of dead phytoplankton was insufficient to sustain them^17^. In addition, Bacteria that thrived on detritus could strongly influence the food web, as Bacteria are intensively consumed by Protozoa, which in turn were eaten by higher trophic levels^13^. Since the average depth of Palude della Rosa is about 0.5 m^34^, there is a close coupling between the benthic and pelagic environments^29^, so sediment resuspension has profoundly altered the ecosystem, not only because of greater resources for detritivores^75^, but also because of the resuspension of benthic FNs^11,31^. These accounted for most of the total primary production, especially in 2007 when the primary production of benthic FNs was more than three times that of pelagic FNs (Table 1).

A hypothetical persistence of conditions, such as those highlighted in our work, would inevitably have implications for the population dynamics of consumers at higher trophic levels, and thus for the structure and functioning of entire food webs^77–79^. In fact, a sharp decline in the abundance of fish feeding on plankton was observed in the Venice Lagoon landings from 1995 to 2001, with a decrease in the ratio between pelagic and demersal fishes that showed a value of less than 1, as well as an effect of the rapid decline of higher level consumers^80^. This phenomenon might appear to be overfishing, but instead it was due to the direct and indirect impact of Manila clam harvesting on the entire ecosystem^80^.

The approach used in this study proved to be a valid tool for capturing and interpreting the major forces affecting aquatic food web dynamics at two different time points even in highly dynamic environments. However, the application of this network approach to consecutive plankton samples in time and space is necessary in the future to link them to possible interpretations or predictions of future scenarios. With the latter in mind, and measures to identify tools to mitigate and possibly prevent the various sources of impact, the idea was to capture the driving forces of change in lagoon planktonic communities by placing them in the context of the various pressures to which they may be exposed. The results confirm that the plankton community can serve to assess the health of the whole ecosystem^7–9^, as it provided results comparable to those of other studies on high trophic level networks. Indeed, we demonstrated that sediment resuspension was a source of stress^73^ on which the system was highly dependent^75,76^, and that the system increased its resilience at the expense of its efficiency in coping with the perturbation^66^ moving away from its optimum of robustness^51^.

### Supplementary Information

Supplementary Information.

## Data Availability

All data generated or analysed during this study are included in this published article (and its Supplementary Information file).
