# Supplementary material for: Planktonic ecological networks support quantification of changes in ecosystem health and functioning
Source: Sci Rep. 2023 Oct 4;13:16683. doi: 10.1038/s41598-023-43738-y (PMC10550973; doi:10.1038/s41598-023-43738-y)
Supplement: Supplementary file 1 — Supplementary Information. [file 41598_2023_43738_MOESM1_ESM.pdf]

Supplementary Material

Planktonic ecological networks support quantification of changes in ecosystem health and functioning

by

Matteo Loschi, Domenico D'Alelio, Elisa Camatti, Fabrizio Bernardi Aubry, Alfred Beran, Simone Libralato

Methods used to collect original plankton data

Data used to set initial conditions for abundance and biomass were obtained by specific approach for each component of the plankton community. Abundance and Biomass of mixo-dinoflagellates, (Dinoflagellata less than 20 µm) FN 18 (Table 1), comprising species that can use a mix of different sources of energy and carbon, instead of having a single trophic mode, were obtained following the nanophytoplankton methods.

Picophytoplankton (size between 0.2 and 2 µm)

In order to estimate the autotrophic picoplankton abundance, water samples were preserved with pre-filtered buffered formaldehyde, kept in the dark at 4 °C and analyzed within one week. Duplicate slides were prepared from the samples by filtering 5–10 ml of sea water from each sample onto 0.2 µm pore size Nucleopore black membranes. The cell counts were made using a Zeiss Axiovert 35 microscope, equipped with a HBO 50 W light. A BP 450–490 exciter filter, an FT 510 chromatic beam splitter and an LP 520 barrier filter were used. At least 20 randomly selected fields were counted for each slide, at a final ×1000 magnification to determine abundance. Cell sizes of randomly selected individuals were measured by Image analysis, using Image Pro Express (Media Cybernetics). Most cells were coccoid or rod-shaped; in order to determine their carbon biomass, cell volume was calculated according to Tamigneaux et al. (1995)[1].

Heterotrophic picoplankton (size between 0.2 and 2 µm)

10 mL seawater samples were fixed with pre-filtered borate-buffered formalin (0.2 µm) at 2% of the final concentration and stained for 15 min with 4'6-diamidino-2-phenylindole (DAPI, Sigma), final concentration 1 µg ml^{-1} [2]. Samples were filtered in triplicate through 0.2 µm black colored polycarbonate filters (Nucleopore) and stored at -20 °C. The filters were mounted on slides and analyzed under a microscope (Olympus BX 60 F5 epifluorescence microscope at 1000x) and counted under a UV filter set (BP 330–385 nm). A minimum of 300 cells from at least 20 random fields were counted for each filter. The bacterial biomass was obtained by multiplying the bacterial abundance by the conversion factor of 20 fgC $cell^{-1}$ [3].

Nano- (size between 2 and 20 µm) and microphytoplankton (size between 20 and 200 µm)

Nano- and microphytoplankton were fixed in formalin neutralized with hexamethylenetetramine and recognized and counted with an inverted microscope[4–6]. The taxa composition was mainly performed

according to Tomas (1997). At least 200 organisms (often more than 500) were counted for each sample, expressing the abundance in l^{-1} cells. Morphometric measurements of phytoplankton cells were performed by image analysis, with a digital color camera (Go Cameras Go-5). For each species, linear dimensions (µm) were obtained by direct measurement. From these measurements we calculated the biovolume of the organism (V; μm^3) according to Strathmann (1967)[7]. Through the average cell biovolume we calculated the carbon content using the formulas introduced by Smetacek (1975)[8].

Heterotrophic nanoplankton (size between 2 and 20 µm)

The water samples were preserved with glutaraldehyde (10% final concentration) and stained with DAPI at a final concentration of 1 $\mu g\ ml^{-1}$. After filtering 20 ml from each sample through black prestained Nucleopore polycarbonate filters (0.8 µm pore size, 25 mm diameter), duplicate slides were prepared. Cell counts were performed using a Zeiss Axiovert 35 microscope, equipped with a 100 W HBO light, a BP 365/12 exciter filter, an FT 395 chromatic beam splitter and an LP 397 barrier filter. The cell size measurements were done individually. Cell volume was calculated on approximately 200 cells (randomly selected over a variable number of microscopic fields) from a two-dimensional cellular image. The organic carbon values were obtained by multiplying the cell volume by 0.14 as indicated by Edler (1979)[9].

Microzooplankton (size between 20 and 200 µm)

Abundance and biomass of microzooplankton was determined using 2 l samples fixed with hexamethylenetetramine-buffered formaldehyde at a final concentration of 1.5%. After a first step of preconcentration via sedimentation to 200 ml, at least 50 ml were settled in sedimentation chambers and counted following the method according to Utermöhl (1958)[4]. Samples were examined with a ZEISS IM135 inverted microscope at 200x. For the determination of the biomass cells were measured and grouped according to standardized geometrical forms. Cell volume was transformed into carbon biomass using the conversion factor of 14 pg C μm^{-3} [10] for aloricates and the formula 444.5 pg C + (lorica volume in μm^{-3} x 0.053 pg C) for loricate ciliates[11].

Meso- (size between 0.20 and 20 mm) and macrozooplankton (size between 2 and 20 mm)

Meso- and macrozooplankton samples were collected at the surface (0.5 m depth) by an Apstein standard net (diameter 0.4 m), equipped with a flowmeter (HydroBios) to measure the volume of water filtered. Samples were preserved in formaldehyde (4% final concentration). In the laboratory, the organisms were subsampled and at least two subsamples were counted completely and identified, according to the International Council for the Exploration of the Sea (ICES) protocol to species level, where possible, or to higher taxonomic levels[12].

The meso- and macrozooplankton averaged Carbon-biomasses were estimated by multiplying the average abundance of each FN i) the carbon-per-individual quota for the same FNs as derived from published data[13–17]; and ii) the vertical extension of the water-layer within which these organisms were sampled at Palude della Rosa (about 0.5 m). In case of multi-species FNs (44-46), the average carbon per individual was estimated taking into account the quantitative proportion of each species based on not-aggregated abundance data.

Non-living groups

The total amount of non-living organic matter was estimated by subtracting the total amount of biomass allocated within unicellular plankton biomass from the POC. The biomass of the two different categories of faecal pellets (i.e., size less or greater than 200 µm in size) was estimated with the formula:

$$B_{\text{faecal pellets}} = B_{\text{animal}} \cdot \alpha_{\text{animal}} \cdot \varepsilon_{\text{animal}}$$

where α and ε were the consumption rate per biomass unit and the unassimilated fraction of biomass consumed by the animal, respectively. More details can be found in D'Alelio et al. (2016)[14].

Bibliography

1. Tamigneaux, E., Vazquez, E., Mingelbier, M., Klein, B. & Legendre, L. Environmental control of phytoplankton assemblages in nearshore marine waters, with special emphasis on phototrophic ultraplankton. *J. Plankton Res.* **17**, 1421–1448 (1995).
2. Porter, K. G. & Feig, Y. S. The use of DAPI for identifying and counting aquatic microflora. *Limnol. Oceanogr.* **25**, 943–948 (1980).
3. Lee, S. & Fuhrman, J. A. Relationships between Biovolume and Biomass of Naturally Derived Marine Bacterioplankton. *Appl. Environ. Microbiol.* **53**, 1298–1303 (1987).
4. Utermöhl, H. Zur Vervollkommnung der quantitativen Phytoplankton-Methodik. *Mitteilungen - Int. Vereinigung für Theor. und Angew. Limnol.* **9**, 1–38 (1958).
5. Throndsen, J. Preservation and storage. in *Monographs on Oceanographic Methodology 6. Phytoplankton Manual* (ed. Sournia, A.) 69–74 (Unesco, 1978). doi:10.1002/iroh.19800650312
6. Zingone, A. *et al.* Fitoplancton: metodiche di analisi quali-quantitativa. in *Metodologie di campionamento e di studio del plancton marino* (eds. Socal, G. et al.) 213–237 (ISPRA, 2010).
7. Strathmann, R. R. ESTIMATING THE ORGANIC CARBON CONTENT OF PHYTOPLANKTON FROM CELL VOLUME OR PLASMA VOLUME. *Limnol. Oceanogr.* **12**, 411–418 (1967).
8. Smetacek, V. Die Sukzession des Phytoplanktons in der westlichen Kieler Bucht. (Kiel University, 1975).
9. Edler, L. *Recommendations on Methods for Marine Biological Studies in the Baltic Sea: Phytoplankton and chlorophyll.* (The Baltic Marine Biologists, 1979).
10. Putt, M. & Stoecker, D. K. An experimentally determined carbon : volume ratio for marine "oligotrichous" ciliates from estuarine and coastal waters. *Limnol. Oceanogr.* **34**, 1097–1103 (1989).
11. Verity, P. G. & Langdon, C. Relationships between lorica volume, carbon, nitrogen, and ATP content of tintinnids In Narragansett Bay. *J. Plankton Res.* **6**, 859–868 (1984).
12. *ICES Zooplankton Methodology Manual*. (Academic Press, 2000). doi:10.1016/B978-0-12-327645-2.X5000-2
13. Almeda, R. *et al.* Trophic role and carbon budget of metazoan microplankton in northwest Mediterranean coastal waters. *Limnol. Oceanogr.* **56**, 415–430 (2011).
14. D'Alelio, D., Libralato, S., Wyatt, T. & Ribera d'Alcalà, M. Ecological-network models link diversity, structure and function in the plankton food-web. *Sci. Rep.* **6**, 21806 (2016).
15. Riccardi, N. Selectivity of plankton nets over mesozooplankton taxa: implications for abundance, biomass and diversity estimation. *J. Limnol.* **69**, 287–296 (2010).
16. Robert, D., Castonguay, M. & Fortier, L. Effects of intra- and inter-annual variability in prey field on the feeding selectivity of larval Atlantic mackerel (*Scomber scombrus*). *J. Plankton Res.* **30**, 673–688 (2008).
17. Heidelberg, K. B., O'Neil, K. L., Bythell, J. C. & Sebens, K. P. Vertical distribution and diel patterns of zooplankton abundance and biomass at Conch Reef, Florida Keys (USA). *J. Plankton Res.* **32**, 75–91 (2010).

Supplementary Data

Maximum and minimum input values of metabolic parameters (μ is the production rate per biomass unit, α is the consumption rate per biomass unit, ε is the unassimilated fraction of biomass consumed, and ph is the phototrophy proportion in individual metabolism) in 2005 and 2007 for each functional node (FN)

2005

**Maximum values**	FN	μ (d^{-1})	α (d^{-1})	ε	ph		**Minimum values**	FN	μ (d^{-1})	α (d^{-1})	ε	ph
**Living nodes**	1	1,2	0	0,01	1,000		**Living nodes**	1	0,6	0	0,01	1,000
	6	1,3	0	0,01	1,000			6	0,7	0	0,01	1,000
	8	1,59	0	0,01	1,000			8	0,8	0	0,01	1,000
	9	1,46	0	0,01	1,000			9	0,7	0	0,01	1,000
	10	2,15	0	0,01	1,000			10	1,1	0	0,01	1,000
	14	1,39	0	0,01	1,000			14	0,7	0	0,01	1,000
	16	1,5	0	0,01	1,000			16	0,8	0	0,01	1,000
	17	1,9	0	0,01	1,000			17	1	0	0,01	1,000
	23	1,46	0,09	0,01	0,950			23	0,7	0,05	0,01	0,950
	24	0,98	4,7	0,01	0,000			24	0,5	2,35	0,01	0,000
	26	0,875	3,5	0,01	0,000			26	0,9	3,5	0,01	0,000
	28	2,15	3	0,01	0,300			28	1,1	1,5	0,01	0,300
	29	2,04	3,84	0,01	0,000			29	1	1,92	0,01	0,000
	32	1,66	7,3	0,01	0,000			32	0,8	3,65	0,01	0,000
	33	1,66	7,3	0,01	0,000			33	0,8	3,65	0,01	0,000
	34	1,76	1,8	0,01	0,000			34	0,9	0,9	0,01	0,000
	35	1,68	7	0,2	0,000			35	0,1	0,7	0,2	0,000
	36	1,66	8,05	0,27	0,000			36	0,2	0,81	0,27	0,000
	37	0,42	2,03	0,27	0,000			37	0	0,2	0,27	0,000
	38	1,37	8,98	0,55	0,000			38	0,1	0,9	0,3	0,000
	39	1,37	8,82	0,55	0,000			39	0,1	0,88	0,3	0,000
	40	0,27	1,18	0,55	0,000			40	0	0,12	0,45	0,000
	41	0,25	1,31	0,55	0,000			41	0	0,13	0,45	0,000
	42	0,27	1,17	0,55	0,000			42	0	0,12	0,45	0,000
	43	0,09	1,23	0,35	0,000			43	0	0,12	0,24	0,000
	44	0,0075	0,05	0,2	0,000			44	0	0,01	0,18	0,000
	45	0,42	7	0,2	0,000			45	0,1	0,7	0,2	0,000
	47	0,42	7	0,2	0,000			47	0,1	0,7	0,2	0,000
	48	0,29	1,55	0,35	0,000			48	0,1	0,16	0,24	0,000
	49	0,9	3,6	0,01	0,000			49	0,9	3,6	0,01	0,000
**Non-living nodes**	50	0	0	0	-1,00		**Non-living nodes**	50	0	0	0	-1,00
	51	0	0	0	-1,00			51	0	0	0	-1,00
	52	0	0	0	-1,00			52	0	0	0	-1,00
	53	0	0	0	-1,00			53	0	0	0	-1,00

2007

Maximum values	FN	μ (d^{-1})	α (d^{-1})	ε	ph		Minimum values	FN	μ (d^{-1})	α (d^{-1})	ε	ph
	1	1,2	0	0,01	1,000			1	0,6	0	0,01	1,000
	2	1,3	0	0,01	1,000			2	0,7	0	0,01	1,000
	3	1,88	0	0,01	1,000			3	0,9	0	0,01	1,000
	4	1,75	0	0,01	1,000			4	0,9	0	0,01	1,000
	5	1,18	0	0,01	1,000			5	0,6	0	0,01	1,000
	6	1,3	0	0,01	1,000			6	0,7	0	0,01	1,000
	7	1,08	0	0,01	1,000			7	0,5	0	0,01	1,000
	8	1,59	0	0,01	1,000			8	0,8	0	0,01	1,000
	9	1,46	0	0,01	1,000			9	0,7	0	0,01	1,000
	11	2,58	0	0,01	1,000			11	1,3	0	0,01	1,000
	12	1,93	0	0,01	1,000			12	1	0	0,01	1,000
	13	1,59	0	0,01	1,000			13	0,8	0	0,01	1,000
	14	1,39	0	0,01	1,000			14	0,7	0	0,01	1,000
	15	1,69	0	0,01	1,000			15	0,9	0	0,01	1,000
	16	1,5	0	0,01	1,000			16	0,8	0	0,01	1,000
	17	1,9	0	0,01	1,000			17	1	0	0,01	1,000
	18	0,94	2,85	0,01	0,500			18	0,5	1,43	0,01	0,500
	19	0,68	4,88	0,01	0,000			19	0,3	2,44	0,01	0,000
	20	2,36	7,08	0,01	0,000			20	1,2	7,08	0,01	0,000
	21	0,735	3,525	0,01	0,000			21	0,7	3,525	0,01	0,000
	22	1,46	0,09	0,01	0,950			22	0,7	0,05	0,01	0,950
	24	0,98	4,7	0,01	0,000			24	0,5	2,35	0,01	0,000
**Living nodes**	25	0,98	4,7	0,01	0,000		**Living nodes**	25	0,5	2,35	0,01	0,000
	26	0,735	3,525	0,01	0,000			26	0,7	3,525	0,01	0,000
	27	1,74	3,84	0,01	0,000			27	0,9	1,92	0,01	0,000
	28	1,97	3	0,01	0,300			28	1	1,5	0,01	0,300
	29	1,95	3,84	0,01	0,000			29	1	1,92	0,01	0,000
	30	1,59	3	0,01	0,300			30	0,8	1,5	0,01	0,300
	31	1,74	3,84	0,01	0,000			31	0,9	1,92	0,01	0,000
	32	1,66	7,3	0,01	0,000			32	0,8	3,65	0,01	0,000
	33	1,66	7,3	0,01	0,000			33	0,8	3,65	0,01	0,000
	34	1,76	1,8	0,01	0,000			34	0,9	0,9	0,01	0,000
	35	1,68	7	0,2	0,000			35	0,1	0,7	0,2	0,000
	36	1,64	8,74	0,27	0,000			36	0,2	0,87	0,27	0,000
	37	0,41	2,18	0,27	0,000			37	0	0,22	0,27	0,000
	39	1,37	8,82	0,55	0,000			39	0,1	0,88	0,3	0,000
	40	0,17	0,84	0,55	0,000			40	0	0,08	0,45	0,000
	41	0,16	0,93	0,55	0,000			41	0	0,09	0,45	0,000
	42	0,18	0,83	0,55	0,000			42	0	0,08	0,45	0,000
	43	0,08	1,23	0,35	0,000			43	0	0,12	0,24	0,000
	44	0,0075	0,05	0,2	0,000			44	0	0,01	0,18	0,000
	46	0,42	7	0,2	0,000			46	0,1	0,7	0,2	0,000
	47	0,42	7	0,2	0,000			47	0,1	0,7	0,2	0,000
	48	0,22	1,33	0,35	0,000			48	0,1	0,13	0,24	0,000
	49	0,9	3,6	0,01	0,000			49	0,9	3,6	0,01	0,000
**Non-living nodes**	50	0	0	0	-1,00		**Non-living nodes**	50	0	0	0	-1,00
	51	0	0	0	-1,00			51	0	0	0	-1,00
	52	0	0	0	-1,00			52	0	0	0	-1,00
	53	0	0	0	-1,00			53	0	0	0	-1,00

Maximum and minimum input values of proportions of flows to non-living nodes (γ) in 2005 and 2007 for each functional node (FN)

2005

Maximum values	FN	50	51	52	53	SUM		Minimum values	FN	50	51	52	53	SUM
Living nodes	1	0,000	0,000	0,870	0,130	1,000		Living nodes	1	0,000	0,000	0,870	0,130	1,000
	6	0,000	0,000	0,870	0,130	1,000			6	0,000	0,000	0,870	0,130	1,000
	8	0,000	0,000	0,870	0,130	1,000			8	0,000	0,000	0,870	0,130	1,000
	9	0,000	0,000	0,870	0,130	1,000			9	0,000	0,000	0,870	0,130	1,000
	10	0,000	0,000	0,870	0,130	1,000			10	0,000	0,000	0,870	0,130	1,000
	14	0,000	0,000	0,870	0,130	1,000			14	0,000	0,000	0,870	0,130	1,000
	16	0,000	0,000	0,870	0,130	1,000			16	0,000	0,000	0,870	0,130	1,000
	17	0,000	0,000	0,870	0,130	1,000			17	0,000	0,000	0,870	0,130	1,000
	23	0,000	0,000	0,876	0,124	1,000			23	0,000	0,000	0,876	0,124	1,000
	24	0,000	0,000	1,000	0,000	1,000			24	0,000	0,000	1,000	0,000	1,000
	26	0,000	0,000	1,000	0,000	1,000			26	0,000	0,000	1,000	0,000	1,000
	28	0,000	0,000	0,961	0,039	1,000			28	0,000	0,000	0,961	0,039	1,000
	29	0,000	0,000	1,000	0,000	1,000			29	0,000	0,000	1,000	0,000	1,000
	32	0,000	0,000	1,000	0,000	1,000			32	0,000	0,000	1,000	0,000	1,000
	33	0,000	0,000	1,000	0,000	1,000			33	0,000	0,000	1,000	0,000	1,000
	34	0,000	0,000	1,000	0,000	1,000			34	0,000	0,000	1,000	0,000	1,000
	35	0,200	0,000	0,800	0,000	1,000			35	0,200	0,000	0,800	0,000	1,000
	36	0,270	0,000	0,730	0,000	1,000			36	0,270	0,000	0,730	0,000	1,000
	37	0,270	0,000	0,730	0,000	1,000			37	0,270	0,000	0,730	0,000	1,000
	38	0,550	0,000	0,450	0,000	1,000			38	0,300	0,000	0,700	0,000	1,000
	39	0,550	0,000	0,450	0,000	1,000			39	0,300	0,000	0,700	0,000	1,000
	40	0,550	0,000	0,450	0,000	1,000			40	0,450	0,000	0,550	0,000	1,000
	41	0,550	0,000	0,450	0,000	1,000			41	0,450	0,000	0,550	0,000	1,000
	42	0,550	0,000	0,450	0,000	1,000			42	0,450	0,000	0,550	0,000	1,000
	43	0,350	0,000	0,650	0,000	1,000			43	0,240	0,000	0,760	0,000	1,000
	44	0,000	0,200	0,800	0,000	1,000			44	0,000	0,180	0,820	0,000	1,000
	45	0,200	0,000	0,800	0,000	1,000			45	0,200	0,000	0,800	0,000	1,000
	47	0,200	0,000	0,800	0,000	1,000			47	0,200	0,000	0,800	0,000	1,000
	48	0,350	0,000	0,650	0,000	1,000			48	0,240	0,000	0,760	0,000	1,000
	49	0,000	0,000	1,000	0,000	1,000			49	0,000	0,000	1,000	0,000	1,000
Non-living nodes	50	1,000	0,000	0,000	0,000	1,000		Non-living nodes	50	1,000	0,000	0,000	0,000	1,000
	51	0,000	1,000	0,000	0,000	1,000			51	0,000	1,000	0,000	0,000	1,000
	52	0,000	0,000	1,000	0,000	1,000			52	0,000	0,000	1,000	0,000	1,000
	53	0,000	0,000	0,000	1,000	1,000			53	0,000	0,000	0,000	1,000	1,000

2007

Maximum values	FN	50	51	52	53	SUM
Living nodes	1	0,000	0,000	0,870	0,130	1,000
	2	0,000	0,000	0,870	0,130	1,000
	3	0,000	0,000	0,870	0,130	1,000
	4	0,000	0,000	0,870	0,130	1,000
	5	0,000	0,000	0,870	0,130	1,000
	6	0,000	0,000	0,870	0,130	1,000
	7	0,000	0,000	0,870	0,130	1,000
	8	0,000	0,000	0,870	0,130	1,000
	9	0,000	0,000	0,870	0,130	1,000
	11	0,000	0,000	0,870	0,130	1,000
	12	0,000	0,000	0,870	0,130	1,000
	13	0,000	0,000	0,870	0,130	1,000
	14	0,000	0,000	0,870	0,130	1,000
	15	0,000	0,000	0,870	0,130	1,000
	16	0,000	0,000	0,870	0,130	1,000
	17	0,000	0,000	0,870	0,130	1,000
	18	0,000	0,000	0,935	0,065	1,000
	19	0,000	0,000	1,000	0,000	1,000
	20	0,000	0,000	1,000	0,000	1,000
	21	0,000	0,000	1,000	0,000	1,000
	22	0,000	0,000	0,876	0,124	1,000
	24	0,000	0,000	1,000	0,000	1,000
	25	0,000	0,000	1,000	0,000	1,000
	26	0,000	0,000	1,000	0,000	1,000
	27	0,000	0,000	1,000	0,000	1,000
	28	0,000	0,000	0,961	0,039	1,000
	29	0,000	0,000	1,000	0,000	1,000
	30	0,000	0,000	0,961	0,039	1,000
	31	0,000	0,000	1,000	0,000	1,000
	32	0,000	0,000	1,000	0,000	1,000
	33	0,000	0,000	1,000	0,000	1,000
	34	0,000	0,000	1,000	0,000	1,000
	35	0,200	0,000	0,800	0,000	1,000
	36	0,270	0,000	0,730	0,000	1,000
	37	0,270	0,000	0,730	0,000	1,000
	39	0,550	0,000	0,450	0,000	1,000
	40	0,550	0,000	0,450	0,000	1,000
	41	0,550	0,000	0,450	0,000	1,000
	42	0,550	0,000	0,450	0,000	1,000
	43	0,350	0,000	0,650	0,000	1,000
	44	0,000	0,200	0,800	0,000	1,000
	46	0,200	0,000	0,800	0,000	1,000
	47	0,200	0,000	0,800	0,000	1,000
	48	0,350	0,000	0,650	0,000	1,000
	49	0,000	0,000	1,000	0,000	1,000
Non-living nodes	50	1,000	0,000	0,000	0,000	1,000
	51	0,000	1,000	0,000	0,000	1,000
	52	0,000	0,000	1,000	0,000	1,000
	53	0,000	0,000	0,000	1,000	1,000

Minimum values	FN	50	51	52	53	SUM
Living nodes	1	0,000	0,000	0,870	0,130	1,000
	2	0,000	0,000	0,870	0,130	1,000
	3	0,000	0,000	0,870	0,130	1,000
	4	0,000	0,000	0,870	0,130	1,000
	5	0,000	0,000	0,870	0,130	1,000
	6	0,000	0,000	0,870	0,130	1,000
	7	0,000	0,000	0,870	0,130	1,000
	8	0,000	0,000	0,870	0,130	1,000
	9	0,000	0,000	0,870	0,130	1,000
	11	0,000	0,000	0,870	0,130	1,000
	12	0,000	0,000	0,870	0,130	1,000
	13	0,000	0,000	0,870	0,130	1,000
	14	0,000	0,000	0,870	0,130	1,000
	15	0,000	0,000	0,870	0,130	1,000
	16	0,000	0,000	0,870	0,130	1,000
	17	0,000	0,000	0,870	0,130	1,000
	18	0,000	0,000	0,935	0,065	1,000
	19	0,000	0,000	1,000	0,000	1,000
	20	0,000	0,000	1,000	0,000	1,000
	21	0,000	0,000	1,000	0,000	1,000
	22	0,000	0,000	0,876	0,124	1,000
	24	0,000	0,000	1,000	0,000	1,000
	25	0,000	0,000	1,000	0,000	1,000
	26	0,000	0,000	1,000	0,000	1,000
	27	0,000	0,000	1,000	0,000	1,000
	28	0,000	0,000	1,000	0,000	1,000
	29	0,000	0,000	1,000	0,000	1,000
	30	0,000	0,000	1,000	0,000	1,000
	31	0,000	0,000	1,000	0,000	1,000
	32	0,000	0,000	1,000	0,000	1,000
	33	0,000	0,000	1,000	0,000	1,000
	34	0,000	0,000	1,000	0,000	1,000
	35	0,200	0,000	0,800	0,000	1,000
	36	0,270	0,000	0,730	0,000	1,000
	37	0,270	0,000	0,730	0,000	1,000
	39	0,300	0,000	0,700	0,000	1,000
	40	0,450	0,000	0,550	0,000	1,000
	41	0,450	0,000	0,550	0,000	1,000
	42	0,450	0,000	0,550	0,000	1,000
	43	0,240	0,000	0,760	0,000	1,000
	44	0,000	0,180	0,820	0,000	1,000
	46	0,200	0,000	0,800	0,000	1,000
	47	0,200	0,000	0,800	0,000	1,000
	48	0,240	0,000	0,760	0,000	1,000
	49	0,000	0,000	1,000	0,000	1,000
Non-living nodes	50	1,000	0,000	0,000	0,000	1,000
	51	0,000	1,000	0,000	0,000	1,000
	52	0,000	0,000	1,000	0,000	1,000
	53	0,000	0,000	0,000	1,000	1,000
